# Applicability of machine learning methods for classifying lightweight pigs in commercial conditions

**DOI:** 10.1093/tas/txae171

**Published:** 2024-12-05

**Authors:** Pau Salgado-López, Joaquim Casellas, Iara Solar Diaz, Thomas Rathje, Josep Gasa, David Solà-Oriol

**Affiliations:** Department of Animal and Food Science, Animal Nutrition and Welfare Service (SNIBA), Autonomous University of Barcelona, Bellaterra 08193, Spain; Department of Animal and Food Science, Autonomous University of Barcelona, Bellaterra 08193, Spain; DNA Genetics LLC, Columbus, NE 68601, USA; DNA Genetics LLC, Columbus, NE 68601, USA; Department of Animal and Food Science, Animal Nutrition and Welfare Service (SNIBA), Autonomous University of Barcelona, Bellaterra 08193, Spain; Department of Animal and Food Science, Animal Nutrition and Welfare Service (SNIBA), Autonomous University of Barcelona, Bellaterra 08193, Spain

**Keywords:** area under the curve, classification algorithms, efficiency, growth retardation, live weight, swine

## Abstract

The varying growth rates within a group of pigs present a significant challenge for the current all-in-all-out systems in the pig industry. This study evaluated the applicability of statistical methods for classifying pigs at risk of growth retardation at different production stages using a robust dataset collected under commercial conditions. Data from 26,749 crossbred pigs (Yorkshire × Landrace) with Duroc at weaning (17 to 27 d), 15,409 pigs at the end of the nursery period (60 to 78 d), and 4996 pigs at slaughter (151 to 161 d) were analyzed under three different cut points (lowest 10%, 20%, and 30% weights) to characterize light animals. Records were randomly split into training and testing sets in a 2:1 ratio, and each training dataset was analyzed using an ordinary least squares approach and three machine learning algorithms (decision tree, random forest, and generalized boosted regression). The classification performance of each analytical approach was evaluated by the area under the curve (**AUC**). In all production stages and cut points, the random forest and generalized boosted regression models demonstrated superior classification performance, with AUC estimates ranging from 0.772 to 0.861. The parametric linear model also showed acceptable classification performance, with slightly lower AUC estimates ranging from 0.752 to 0.818. In contrast, the single decision tree was categorized as worthless, with AUC estimates between 0.608 and 0.726. Key prediction factors varied across production stages, with birthweight-related factors being most significant at weaning, and weight at previous stages becoming more crucial later in the production cycle. These findings suggest the potential of machine learning algorithms to improve decision-making and efficiency in pig production systems by accurately identifying pigs at risk of growth retardation.

## Teaser

The use of machine learning algorithms on large commercial datasets that are updated regularly could allow the swine industry to identify pigs at risk of growth retardation, thus improving management interventions, enhancing efficiency, and reducing body weight variability at slaughter.

## INTRODUCTION

Body weight (**BW**) variability is a primary factor affecting production efficiency and profitability, presenting a costly problem for the pig industry (Patience et al., 2004). Though it has been studied that slow-growing pigs play a relevant role in this issue, the early and reliable identification of this subset of pigs is often inconsistent. Such pigs are expected to reach target slaughter weights later than their faster-growing counterparts (López-Vergé et al., 2018), which poses a challenge in swine production systems that aim to maintain contemporary pigs in the same batch to ensure optimal animal health and management efficiency (Maes et al., 2004). Calderón Díaz et al. (2017) reported that delaying pigs from the normal production flow in current all-in-all-out systems is negatively associated with pig health and performance. Moreover, significant variability in market weight lowers carcass value and reduces the operational efficiency of slaughterhouse equipment (López-Vergé et al., 2018).

The incidence of pigs at risk of growth retardation has increased over the past decade, primarily due to ongoing genetic advancements that have increased litter sizes at birth. Consequently, a higher percentage of piglets are born with low birth weights (Beaulieu et al., 2010), contributing to increases of up to 25% in BW variability at birth, as measured by the coefficient of variation (López-Vergé et al., 2018). Although pigs’ BW categories during the initial growth phases can change (Blavi et al., 2021), and the implementation of different management and nutritional practices (Patience et al., 2004; Solà-Oriol and Gasa, 2017) tends to reduce variability with age, it is estimated that 10% to 15% of pigs in any given batch remain classified as slow-growing (Calderón Díaz et al., 2017). Identifying these pigs is crucial for optimizing efficiency and profitability in the swine industry.

Artificial intelligence is a field that integrates computer science and large datasets to develop intelligent systems capable of performing tasks typically requiring human intelligence (Sarker, 2022). Machine learning, a branch of artificial intelligence, utilizes subsets of data to generate algorithms that use different combinations of features, enabling the prediction of outputs based on what the algorithm has learned from the data (Choi et al., 2020). Many machine learning approaches have been developed since the early 1960s, with significant impact on various types of problems, such as classification (Nayeri et al., 2019). Therefore, the application of artificial intelligence in the animal production field is not new (Firk et al., 2002; Rorie et al., 2002). The advancements in computer performance in recent decades have accelerated the pace of development of several methodologies (Curti et al., 2023) with potential contributions to the swine industry. The development of robust tools to identify slow-growing pigs, along with identifying potential risk factors associated with their occurrence, will help develop effective strategies to ensure homogeneity and optimal growth among pigs. Casellas et al. (2024) stated that machine learning algorithms applied to a large dataset from a pure-bred nucleus provide reliable alternatives to parametric linear models for analyzing continuous traits. However, the ability of these algorithms to identify pigs at risk of growth retardation under commercial conditions has not been tested.

Therefore, the objective of this research was to evaluate the applicability of machine learning algorithms for classifying light pigs at each phase within the pig production cycle, using a powerful and robust dataset collected from a single commercial breeding herd. Specifically, we aimed to 1) classify pigs that would be the main contributors to variation within a population and 2) identify the main production factors that explain low pig BW at each stage of production.

## MATERIALS AND METHODS

Approval from the Animal Care and Use Committee was not required for this study, as the analyses were conducted on existing field data collected under standard farm management practices from a commercial farm operated by Andrimner Genética Aplicada (Barcelona, Spain) and DNA Genetics (Columbus, NE, USA).

### Commercial Pig Farm Data

All data were collected from a single farm in the United States between March 2019 and May 2023, focusing on Line 246 (Yorkshire × Landrace) × Duroc (DNA Genetics). The animals were housed under standard intensive farming conditions and were individually weighed at the following stages: birth (**BIW**), weaning (**WW**, 17 to 27 d), the end of the nursery period (60 to 78 d), and slaughter (151 to 161 d). Three different cut points were established at each production stage, corresponding to the lowest 10%, 20%, and 30% of weights. These thresholds were selected because they effectively represent light pigs, as supported by studies (Douglas et al., 2014; He et al., 2016; Montoro et al., 2020), which indicate that pigs in the lower quartile of the BW population distribution typically require additional time to reach target slaughter weights and are often classified as slow-growing pigs. Moreover, although identifying a smaller percentage of very light pigs can be a useful statistical approach, it may have a limited impact on improving the overall homogenization of the batch. Therefore, the cut points at weaning were 4.31 kg, 4.90 kg, and 5.33 kg; at the end of the nursery period, they were 16.00 kg, 18.20 kg, and 19.80 kg; and at slaughter, they were 92.80 kg, 100.00 kg, and 105.00 kg. These cut points can be adjusted according to the specific needs of each user. Age-related differences at each weigh point were corrected for WW, BW at the end of the nursery period, and slaughter BW using a standard linear adjustment based on total data at 21 d, 65 d, and 155 d, respectively (Table 1). Trained farm staff systematically collected additional data for each piglet (including sex, cause and date of death, cross-fostering date, and adoption litter after cross-fostering, if any), for each sow (parity number), and for each litter (birth date, litter size at birth and after cross-fostering, if any).

**Table 1.** Summary of weaning BW, BW at the end of the nursery period, and slaughter BW in the (Yorkshire × Landrace) × Duroc pig population

	Weaning BW, kg		BW at the end of the nursery period, kg		Slaughter BW, kg	
	*n*	Mean ± SE	*n*	Mean ± SE	*n*	Mean ± SE
Overall	26,749	6.01 ± 0.008	15,409	22.18 ± 0.037	4,996	110.82 ± 0.196
Sex						
Male	13,539	6.07 ± 0.011	7,668	22.13 ± 0.053	2,447	112.83 ± 0.279
Female	13,210	5.96 ± 0.011	7,741	22.23 ± 0.052	2,549	108.89 ± 0.270
Cross-fostered						
No	25,871	6.01 ± 0.008	14,619	22.17 ± 0.038	4,748	110.78 ± 0.201
Yes	878	6.04 ± 0.043	790	22.49 ± 0.158	248	111.49 ± 0.864
Sow parity						
1	9,042	5.68 ± 0.012	6,306	22.11 ± 0.057	3,234	111.94 ± 0.225
2	7,134	6.27 ± 0.016	4,376	22.21 ± 0.070	1,459	111.15 ± 0.386
3	4,865	6.11 ± 0.020	2,341	21.97 ± 0.103	297	97.32 ± 0.777
4	3,433	6.19 ± 0.024	1,220	22.23 ± 0.133	6	96.77 ± 4.051
>4	2,275	6.12 ± 0.028	1,166	22.87 ± 0.130		

### Operational Models

The prediction of WW data was based on the following model,


$$\begin{array}{lll} {\text{W}W}_{ijklmnop}=\; & {\text{B}IW}_{i}+{\text{S}ex}_{j}+{\text{C}ross\text{-}fostered}_{k}+{\text{D}B}_{i}+{\text{D}C}_{i}+{\text{V}B}_{l}+{\text{V}C}_{l}\; & +{\text{S}tillbirths}_{m}+{\text{P}arity\;number}_{n}+{\text{L}itter\;size}_{o}+{\text{S}eason}_{p} \end{array}$$

where BIW_*i*_ represented the birth BW of the *i*th piglet, Sex_*j*_ denoted the sex of the *i*th piglet (male or female), and Cross-fostered_*k*_ indicated whether the piglet was cross-fostered or not. **DB**_*i*_ and **DC**_*i*_ were the differences between the piglet’s BIW and the average litter weight at birth and after cross-fostering, respectively. **VB**_*l*_ and **VC**_*l*_ were the within-litter variances of BW at birth and after cross-fostering, respectively. Stillbirths_*m*_ referred to the number of stillbirths in the litter (0, 1, 2, 3 or >3), Parity number_*n*_ represented the sow’s parity number (1, 2, 3, 4 or >4), Litter size_*o*_ indicated the litter size after cross-fostering (<10, 10, 11, 12, 13, 14, 15, 16, 17 or >17), and Season_*p*_ specified the season of birth (winter, spring, summer, autumn). In addition to the described variables, the model for analyzing BW at the end of the nursery period_*ijklmnop*_ also included WW_*i*_ as a predictor, while the model for analyzing slaughter BW_*ijklmnop*_ included both WW_*i*_ and BW at the end of the nursery period_*i*_ as predictors. Weight data were predicted by considering the inherent systematic effects linked to each pig individually. Thus, the prediction of WW_*ijklmnop*_, BW at the end of the nursery period_*ijklmnop*_, and slaughter BW_*ijklmnop*_ in relation to the upper threshold for light pigs served as the outcome variables in these operational models, which were designed to develop multiclass classification algorithms for identifying lightweight pigs at each stage of the pig production cycle.

The statistical importance of the prediction factors was evaluated by measuring the performance improvement associated with each attribute’s split point, adjusted for the number of observations assigned to the corresponding node. The Gini index served as the performance metric for this assessment. Partial dependence plots were examined for factors accounting for more than 5% importance when classifying light pigs at weaning, the end of the nursery period, and slaughter.

### Weighting Function

The same weighting function (Ψ^-δ/100^) applied by Casellas et al. (2024) was used in this study to appropriately adjust the weighting of all recorded data and mitigate any biases. In this context, Ψ represented the variables WW, BW at the end of the nursery period, and slaughter BW. The parameter δ started from δ = 0 and increased by 1 until the relative weight of the average Ψ minus two standard deviations was 10 times larger than the relative weight of the average Ψ.

The analyses were repeated 1,000 times (for WW, BW at the end of the nursery period, and slaughter BW data) to achieve the necessary number of iterations for the iterative process. While this repetition count exceeded the minimum required in some cases to assess the stability and variability of the results under different weight adjustments, it was standardized across all analyses to ensure consistent model fitting and to generate the corresponding receiver operating characteristic (**ROC**) curves. This approach aimed to strike a balance between computational efficiency and the need for robust, comprehensive ROC curve generation.

### Analytical Approaches and Model Hyperparameter Optimization

All analyses were performed with open-source software R v.4.4.0 (R Core Team, 2024) following the analytical approaches established by Casellas et al. (2024), with specific adjustments for the commercial dataset. In a parametric framework, a weighted linear model was fitted by ordinary least squares (*lm* function, *stats* package), with all variables considered continuous except for Stillbirths_*m*_, Parity number_*n*_, Litter size_*o*_, and Season_*p*_. Three different machine learning approaches of varying complexity were also implemented. From lower to higher complexity, we fitted a decision tree (*rpart* package, *anova* method; Quinlan, 1987), a random forest with 1,000 trees (*ranger* package; Marvin and Ziegler, 2017), and a boosting approach, specifically the generalized boosted regression model (*gbm* package; Friedman, 2001). The boosting approach was set with the following parameters: 1000 trees, a maximum interaction depth of 6, and shrinkage set to 0.01.

The hyperparameters for the machine learning models were tuned following the suggestions of Elith et al. (2008) and Casellas et al. (2024) and were standardized across all approaches to provide similar starting points. This methodology provided a consistent framework for comparative purposes. The random forest and generalized boosted regression models were ultimately fitted with 1,000 trees. Preliminary tests with alternative values (i.e., 100, 500, 5,000, 10,000 trees) indicated that fewer than 1,000 trees did not consistently ensure an adequate model fit, while using more than 1,000 trees yielded only marginal improvements in performance. Although the 1,000-tree configuration slightly exceeded the predicted optimal number of trees based on minimum cross-validation error in all cases and entailed higher computational costs, it was necessary to guarantee a reliable model fit across all scenarios. The maximum interaction depth for each tree in the generalized boosted regression models was set to 6, enabling the capture of complex decision boundaries. The models were initially tested and compared with alternative values (i.e., 3, 5, 7, 9) for interaction depth. While the differences in performance across the tested values in all datasets were almost null, the area under the curve (**AUC**) matched the second decimal place starting from an interaction depth of 6 and higher. A shrinkage parameter of 0.01 was selected after testing different alternatives (i.e., 0.1, 0.05, 0.001, 0.005) in the generalized boosted regression models. This value ensured optimal model fit in all cases, as the AUC for WW, BW at the end of the nursery period, and slaughter BW remained consistent beyond the fourth decimal place when comparing 0.01 with smaller shrinkage parameters. The minimum number of observations per terminal node was set to the default value of 10 in the *gbm* package, as it provided a suitable balance between model complexity and generalization.

### Cross-Validation for Classification Ability

Each model was evaluated using a 3-fold cross-validation method. The dataset was randomly divided into three equal and balanced subsets (33.3% each). Two subsets were combined to form the training dataset for fitting the model, and its classification ability was evaluated against the remaining subset (referred to as the testing dataset). Each training dataset was analyzed using an ordinary least squares approach and three machine learning algorithms, applied under three different cut points (lowest 10%, 20%, and 30% weights) for each phase within the pig production cycle. A total of 1,000 individual repetitions of the 3-fold cross-validation method were performed for each approach. While the training and testing datasets remained constant across iterations, different weights were applied to the models in each repetition, controlled by the weighting function. Once the upper threshold for light pigs (Ψ_*t*_) was defined, the analysis was conducted for each value resulting from this threshold. Classification ability was evaluated using the AUC of the ROC curve, which plots sensitivity against 1—specificity, following the methodology outlined by Casellas et al. (2024). Sensitivity, defined as the proportion of light pigs correctly classified, and specificity, defined as the proportion of nonlight pigs correctly classified, were calculated for each repetition. Both metrics are standard parameters for assessing model performance. The classification performance of a model, as measured by the AUC, can generally be categorized as follows: worthless (<0.7), acceptable (0.7 to 0.8), excellent (0.8 to 0.9), and outstanding (>0.9) (Hosmer and Lemeshow, 2000).

## RESULTS

### Comparison of Classification Ability Across Models

The parametric linear model achieved intermediate classification performance compared to the machine learning approaches, regardless of the cut point assumed for WW (AUC estimates ranging from 0.786 to 0.818; Table 2) and for older stages (AUC estimates ranging from 0.752 to 0.801; Tables 3 and 4). The single decision tree provided the lowest AUC estimates for each phase within the pig production cycle, with values ranging from 0.608 to 0.652 for WW (Table 2) and from 0.638 to 0.726 for older stages (Tables 3 and 4). In contrast, the random forest and generalized boosted regression approaches demonstrated acceptable to excellent classification performance, achieving the highest AUC values in all scenarios (Tables 2, 3, and 4).

**Table 2.** AUC for four classification models used as discrimination tools to identify light pigs at weaning (i.e., live weight standardized to 21 d of age), evaluated using three different weight cut points

	Testing data set			
	1	2	3	Mean ± SE
Cut point < 4.31 kg (10%)				
Linear model	0.823	0.823	0.809	0.818 ± 0.005
Decision tree	0.675	0.604	0.638	0.639 ± 0.021
Random forest	0.829	0.823	0.827	0.826 ± 0.002
Generalized boosted regression	0.837	0.833	0.827	0.832 ± 0.003
Cut point < 4.90 kg (20%)				
Linear model	0.812	0.807	0.802	0.807 ± 0.003
Decision tree	0.622	0.647	0.686	0.652 ± 0.019
Random forest	0.815	0.816	0.817	0.816 ± 0.001
Generalized boosted regression	0.819	0.818	0.816	0.818 ± 0.001
Cut point < 5.33 kg (30%)				
Linear model	0.791	0.782	0.784	0.786 ± 0.003
Decision tree	0.685	0.526	0.612	0.608 ± 0.046
Random forest	0.800	0.801	0.792	0.798 ± 0.003
Generalized boosted regression	0.801	0.796	0.792	0.796 ± 0.003

**Table 3.** AUC for four classification models used as discrimination tools to identify light pigs at the end of the nursery period (i.e., live weight standardized to 65 d of age), evaluated using three different weight cut points

	Testing data set			
	1	2	3	Mean ± SE
Cut point < 16.00 kg (10%)				
Linear model	0.765	0.756	0.790	0.770 ± 0.010
Decision tree	0.716	0.682	0.609	0.669 ± 0.032
Random forest	0.807	0.787	0.804	0.799 ± 0.006
Generalized boosted regression	0.811	0.792	0.820	0.808 ± 0.008
Cut point < 18.20 kg (20%)				
Linear model	0.781	0.767	0.787	0.778 ± 0.006
Decision tree	0.714	0.612	0.664	0.663 ± 0.029
Random forest	0.806	0.793	0.804	0.801 ± 0.004
Generalized boosted regression	0.807	0.793	0.804	0.801 ± 0.004
Cut point < 19.80 kg (30%)				
Linear model	0.755	0.742	0.760	0.752 ± 0.005
Decision tree	0.657	0.651	0.606	0.638 ± 0.016
Random forest	0.780	0.770	0.778	0.776 ± 0.003
Generalized boosted regression	0.778	0.761	0.776	0.772 ± 0.005

**Table 4.** AUC for four classification models used as discrimination tools to identify light pigs at slaughter (i.e., live weight standardized to 155 d of age), evaluated using three different weight cut points

	Testing data set			
	1	2	3	Mean ± SE
Cut point < 92.80 kg (10%)				
Linear model	0.839	0.746	0.745	0.777 ± 0.031
Decision tree	0.659	0.772	0.626	0.686 ± 0.044
Random forest	0.889	0.849	0.835	0.858 ± 0.018
Generalized boosted regression	0.895	0.863	0.824	0.861 ± 0.019
Cut point < 100.00 kg (20%)				
Linear model	0.806	0.779	0.817	0.801 ± 0.011
Decision tree	0.707	0.756	0.714	0.726 ± 0.015
Random forest	0.842	0.848	0.858	0.849 ± 0.005
Generalized boosted regression	0.850	0.856	0.865	0.857 ± 0.004
Cut point < 105.00 kg (30%)				
Linear model	0.782	0.760	0.799	0.780 ± 0.011
Decision tree	0.730	0.645	0.739	0.705 ± 0.030
Random forest	0.822	0.836	0.845	0.834 ± 0.007
Generalized boosted regression	0.820	0.816	0.840	0.825 ± 0.007

**Figure 1.**
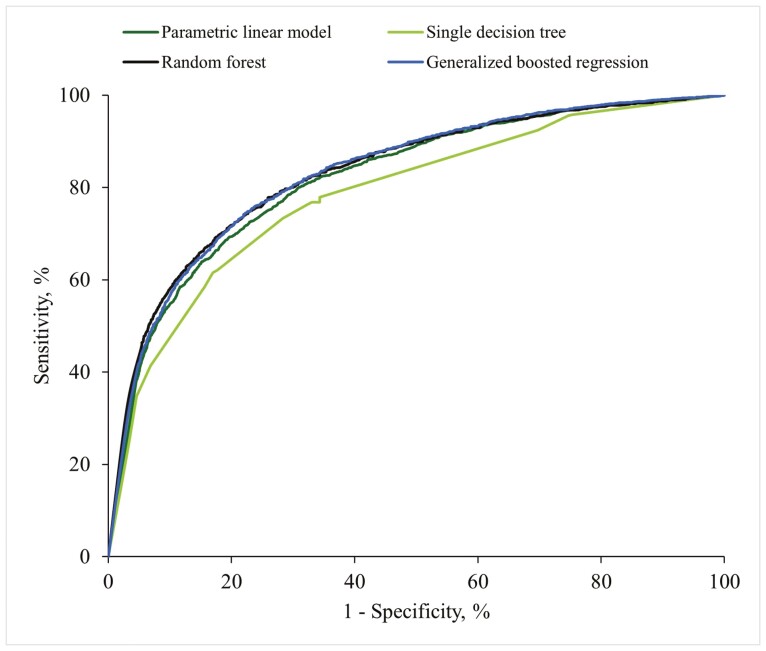
ROC curve for the classification of light pigs at weaning (<4.90 kg) calculated by a parametric linear model and three machine learning procedures (single decision tree, random forest, and generalized boosted regression).

The generalized boosted regression achieved slightly higher AUC estimates than the random forest for the lowest cut points (20% and 10%). At weaning, the AUC increased by 0.25% (cut point, 4.90 kg) and 0.73% (cut point, 4.31 kg), while at older stages, the advantage ranged from 0.00% to 1.13%. The random forest exhibited the best classification performance for the highest cut point (30%), with AUC estimates increasing by 0.75% at weaning, and by 0.52% to 1.10% for older stages, compared with the second highest AUC (i.e., generalized boosted regression). The slight advantage of generalized boosted regression at low specificity values and random forest at high specificity values is shown in Figure 1, where the ROC curves converged only under conditions of nearly complete or null specificity. Furthermore, generalized boosted regression and random forest achieved higher AUC values for smaller cut points for WW, BW at the end of the nursery period, and slaughter BW.

### Importance of Prediction Factors

**Figure 2.**
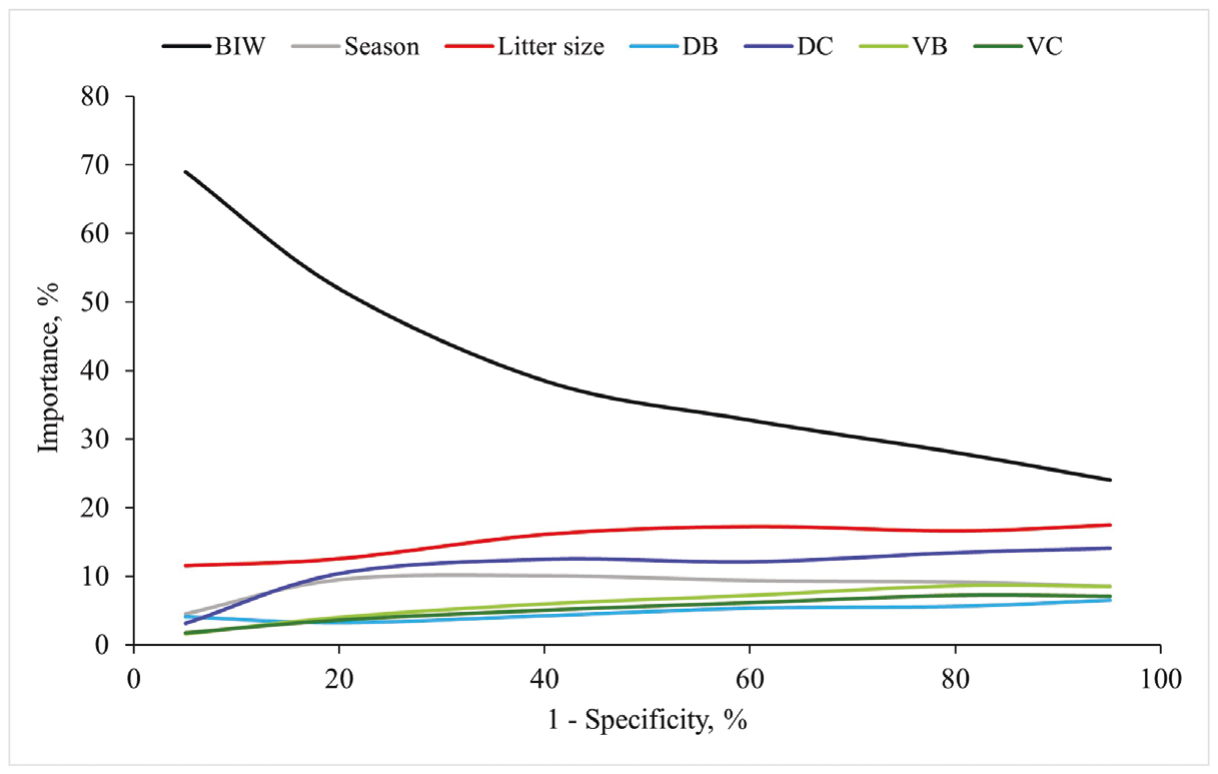
Importance evolution for factors accounting for more than 5% when classifying light pigs at 21 d old (<4.9 kg), with generalized boosted regression. Abbreviations: DB, difference between the piglet’s birth weight and the average litter weight at birth; DC, difference between the piglet’s birth weight and the average litter weight after cross-fostering; BIW, birth weight; VB, within-litter variance of body weight at birth; VC, within-litter variance of body weight after cross-fostering.

**Figure 3.**
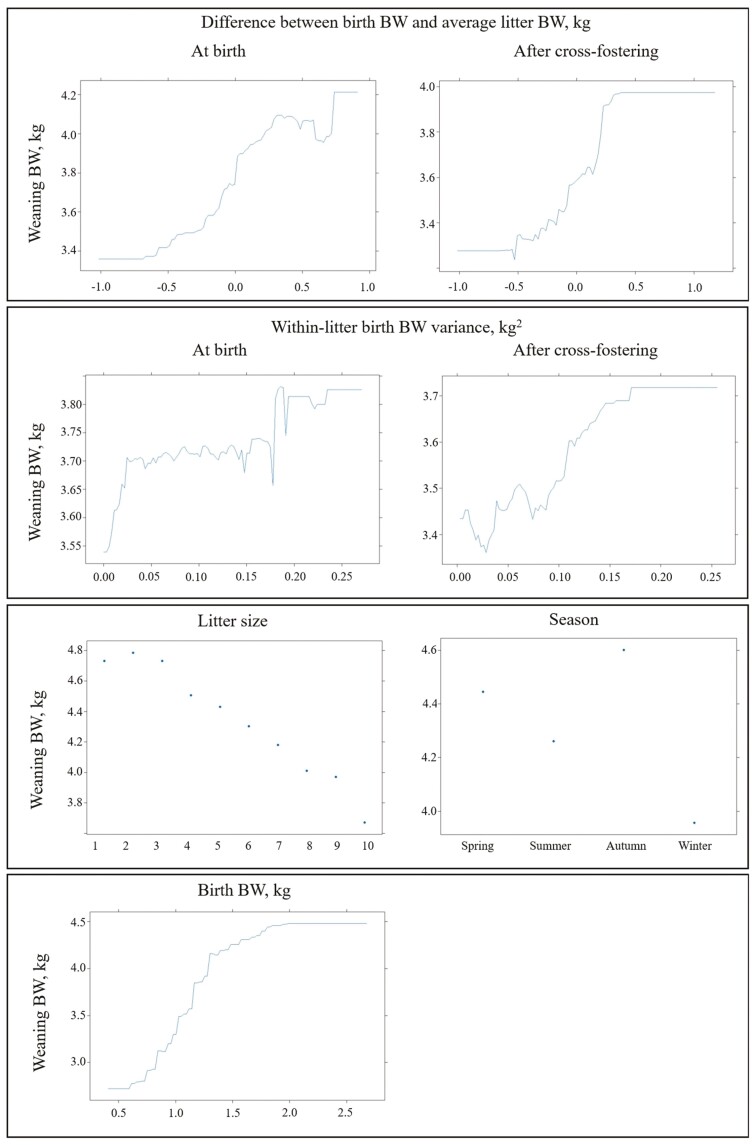
Partial dependence plots for factors accounting for more than 5% importance when classifying light pigs at weaning (<4.9 kg) with generalized boosted regression (sensitivity, 86%; specificity, 60%).

When evaluating the importance of prediction factors for WW analysis, factors BIW_*i*_, Litter size_*o*_, DB_*i*_, DC_*i*_, VB_*l*_, VC_*l*_, and Season_*p*_ individually contributed at least 5% at certain points across the specificity space (Figure 2). The combined importance of all these factors consistently exceeded 85%, with BIW_*i*_ standing out as the most relevant factor, particularly for high specificity values. Additionally, when birthweight-related factors were combined, they explained more than 60% of the specificity space. The predictor Litter size_*o*_ remained the second most important factor along the specificity space. Dependence plots for all these factors were provided in Figure 3.

**Figure 4.**
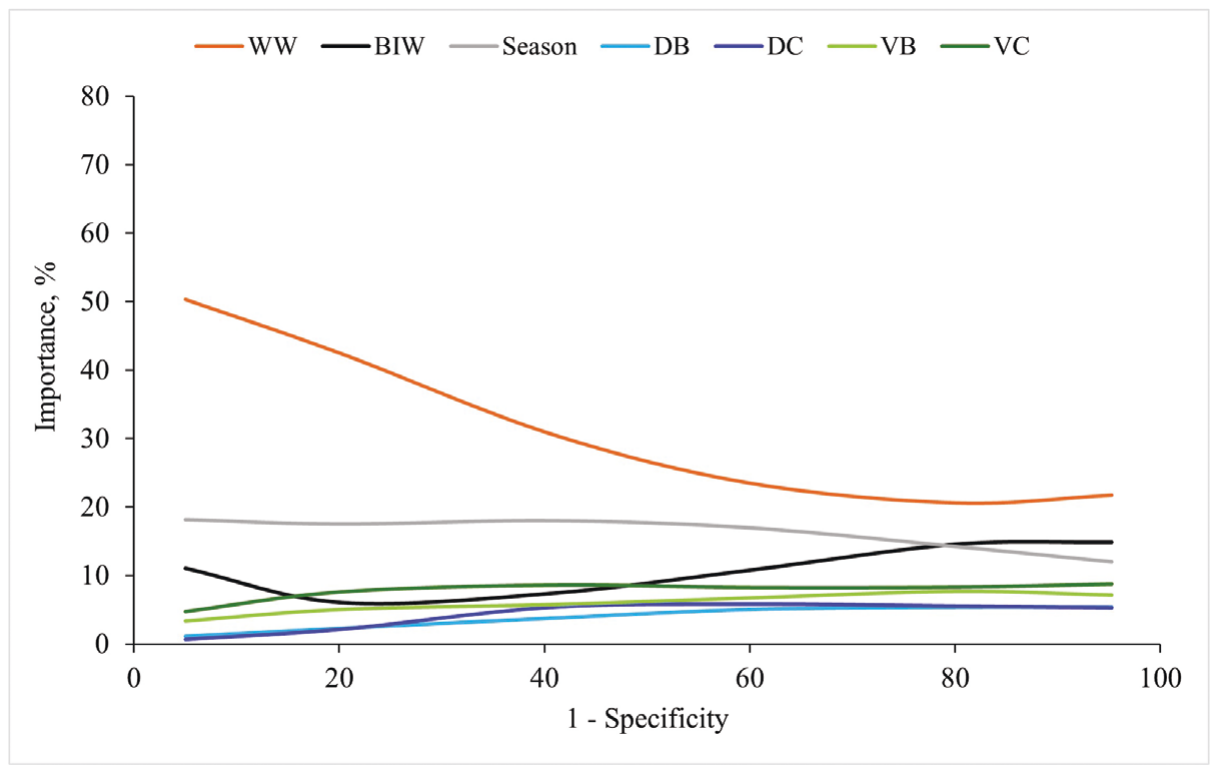
Importance evolution for factors accounting for more than 5% when classifying light pigs at 65 d old (< 18.2 kg), with generalized boosted regression. Abbreviations: DB, difference between the piglet’s birth weight and the average litter weight at birth; DC, difference between the piglet’s birth weight and the average litter weight after cross-fostering; BIW, birth weight; VB, within-litter variance of body weight at birth; VC, within-litter variance of body weight after cross-fostering; WW, weaning weight.

**Figure 5.**
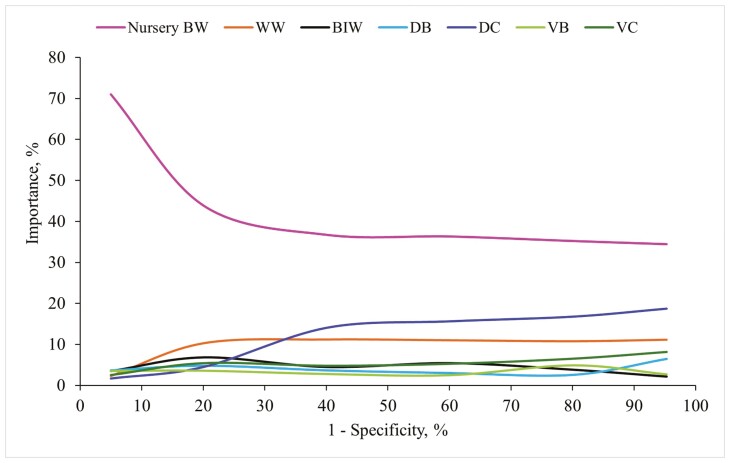
Importance evolution for factors accounting for more than 5% when classifying light pigs at 155 d old (<100.0 kg), with generalized boosted regression. Abbreviations: DB, difference between the piglet’s birth weight and the average litter weight at birth; DC, difference between the piglet’s birth weight and the average litter weight after cross-fostering; BIW, birth weight; Nursery BW, body weight at the end of the nursery period; VB, within-litter variance of body weight at birth; VC, within-litter variance of body weight after cross-fostering; WW, weaning weight.

**Figure 6.**
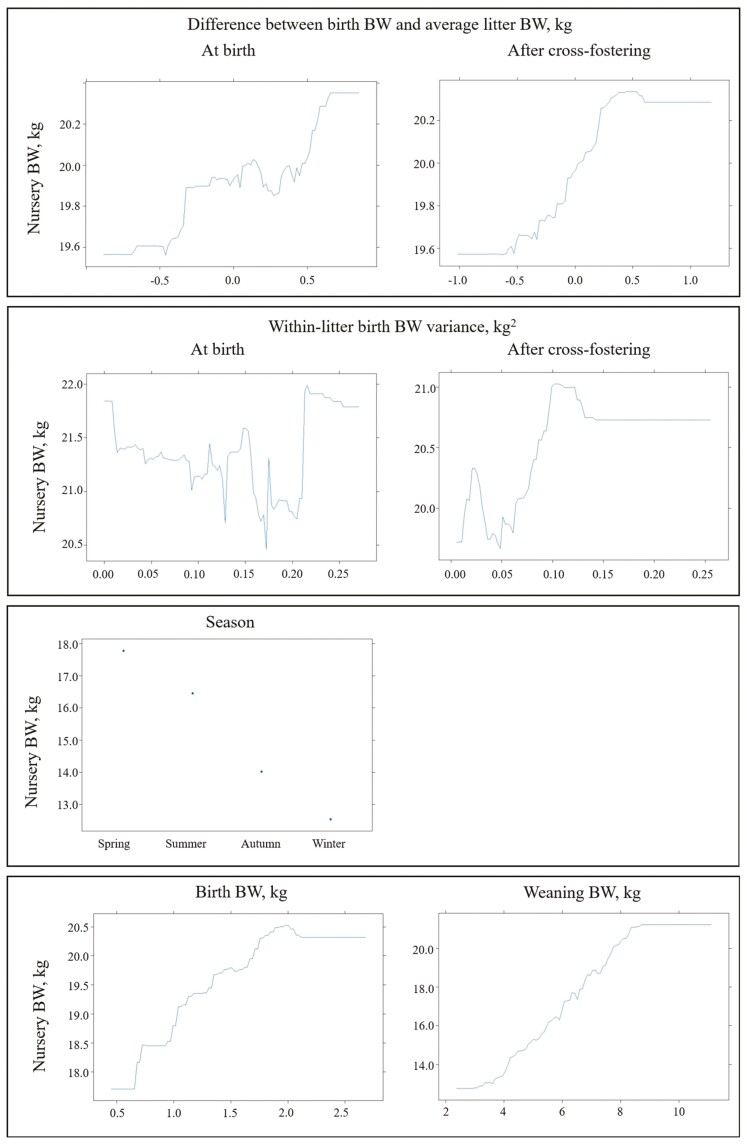
Partial dependence plots for factors accounting for more than 5% importance when classifying light pigs at 65 d old (<18.2 kg) with generalized boosted regression (sensitivity, 85%; specificity, 60%). Abbreviation: Nursery BW, BW at the end of the nursery period.

**Figure 7.**
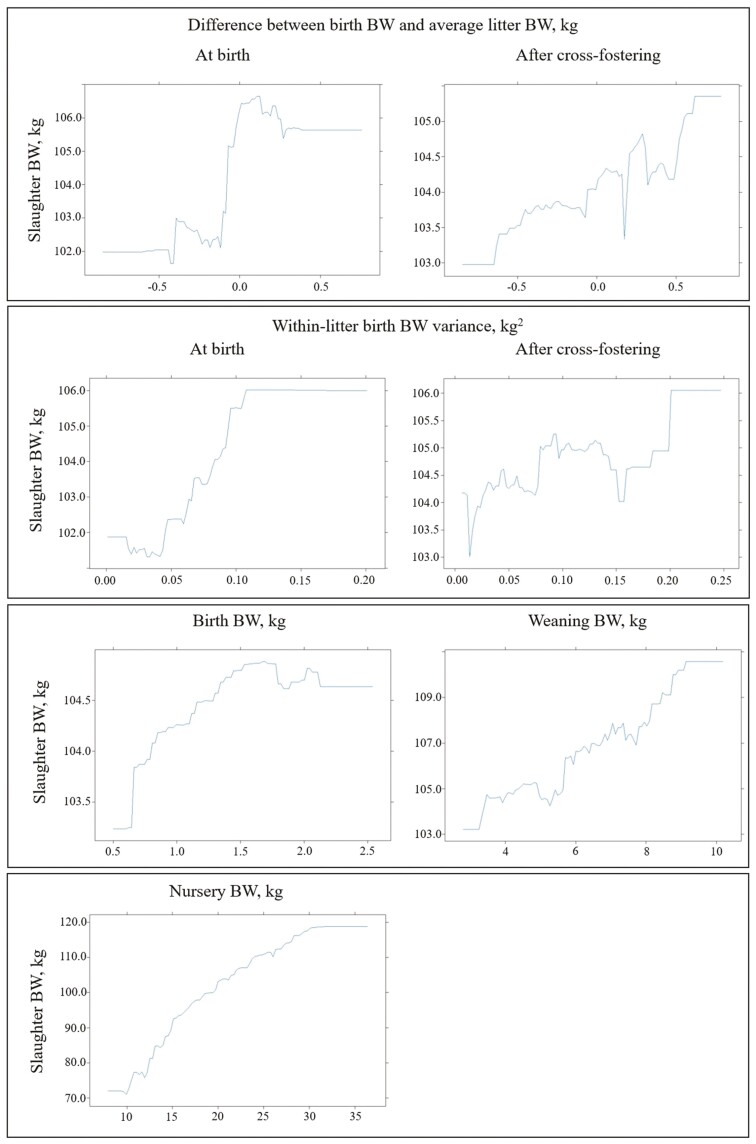
Partial dependence plots for factors accounting for more than 5% importance when classifying light pigs at 155 d old (<100.0 kg) with generalized boosted regression (sensitivity, 90%; specificity, 60%). Abbreviation: Nursery BW, BW at the end of the nursery period.

**Figure 8.**
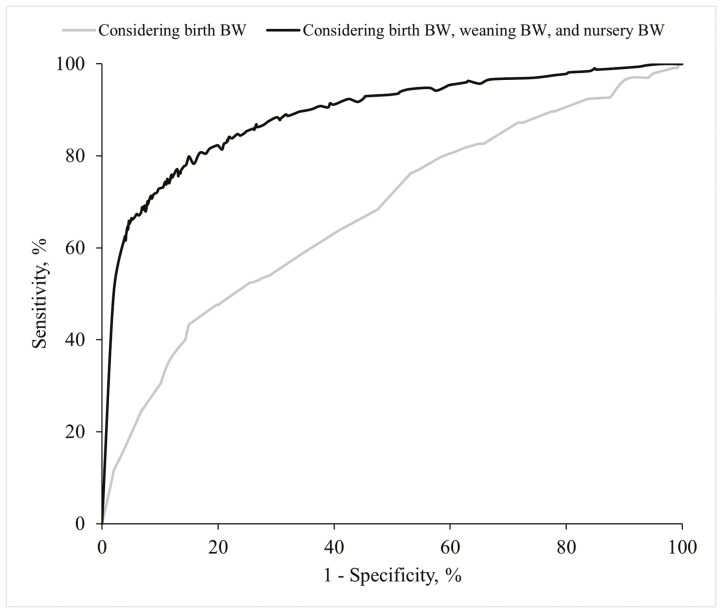
ROC curve for predicting the market BW of (Yorkshire × Landrace) x Duroc pigs based on birth BW, weaning BW, and BW at the end of the nursery period, compared to using only birth BW. Abbreviation: Nursery BW, BW at the end of the nursery period.

In the analysis of BW at the end of the nursery period, the prediction factor WW_*i*_ replaced birthweight-related factors as the most relevant, accounting for more than 50% of the importance for high specificity values (Figure 4). When excluded, the AUC reduced between 0.027 (cut point, 19.80 kg) and 0.040 (cut point, 16.00 kg) (results not shown). Moreover, the prediction factor Season_*p*_ always captured more than 10% of the importance along the specificity space. Similarly, the prediction factor BW at the end of the nursery period_*i*_ was the primary contributor in the analysis of slaughter BW (Figure 5). Excluding this factor from the slaughter BW model resulted in a decrease in AUC ranging from 0.056 (cut point, 92.80 kg) and 0.060 (cut point, 105.00 kg) (results not shown). Dependence plots for factors with more than 5% importance in classifying light pigs at the end of the nursery period and at slaughter were provided in Figures 6 and 7. Furthermore, when the BW for the intermediate phases (weaning and the end of the nursery period) were not considered in predicting slaughter BW, the reduction of the AUC was significant (Figure 8).

## DISCUSSION

Pigs at risk of growth retardation are the main contributors to BW variability throughout the production cycle. It is estimated that, in any given batch, these pigs represent 10% to 15% of the total, leading to inefficient pen utilization and poor carcass grading (Calderón Díaz et al., 2017). Thus, the pig industry would greatly benefit from robust tools to identify these problematic pigs, enabling the development of effective strategies to improve homogeneity and growth within pig production systems.

In recent decades, classification algorithms based on machine learning have emerged as alternative methods with potential applications to the swine industry (Friedman, 2001; Marvin and Ziegler, 2017). In this study, the random forest and generalized boosted regression models achieved the highest classification performance among the four models evaluated. Their classification advantage persisted throughout the ROC curve and was effectively captured by the AUC in our commercial dataset. The AUC values for these models indicated an acceptable to excellent discrimination performance (Hosmer and Lemeshow, 2000), ranging from 0.772 to 0.861. These results are in accordance with findings from Lee et al. (2019) and Casellas et al. (2024), who indicated that machine learning methods can outperform traditional ordinary least squares regression in predicting weight traits in pigs.

In our study, we observed a slight advantage for generalized boosted regression in classifying more extreme pigs, which we attributed to its fine-tuning capability for extreme observations (Friedman, 2001; Chen and Guestrin, 2016). At higher cut points, random forest demonstrated the best classification performance (Su et al., 2022; Casellas et al., 2024). One of the reasons why generalized boosted regression and random forest performed better is their ability to handle heterogeneous features, such as the quantitative and categorical variables in our dataset (Su et al., 2022). Rather than being a disadvantage for this research, the different behavior of these two models depending on the defined cut point should be seen as an opportunity, where the selection of one method over the other can be defined according to the specific needs of each user.

Random forest and generalized boosted regression models demonstrated increased AUC with reduced weight cut points, indicating superior classification performance when focusing on extreme individuals. This result is consistent with previous studies (Steyerberg et al., 2010; Casellas et al., 2024), which have shown that focusing on more extreme cases (i.e., pigs that are much lighter or heavier) increases the difference between the two groups, making them easier to classify. However, regardless of the cut points and the classification models, the differences in classification ability were remarkably small, suggesting minimal benefit from the chosen method and stricter weight cut points. The acceptable to excellent and stable classification performance of random forest and generalized boosted regression across the analytical space is particularly noteworthy for their potential implementation in the swine industry.

In the database of commercial terminal pigs used in this study, the primary influence on the weaning, nursery, and grow-finish stages is best described by the BW at the beginning of each respective period (birth, weaning, and end of the nursery period). As reported by Casellas et al. (2024), the importance of each of these factors decreases throughout the production cycle in favor of the weights recorded at each subsequent stage, indicating that the influence of a prediction factor increases the closer it is to the weight measurement. Consequently, a significant decrease in the AUC of the ROC curves is observed when final marketing weight predictions are based solely on BIW. Several studies have proposed thresholds to identify low BIW piglets (i.e., ≤1.1 kg by Wang et al., 2017; ≤1.15 kg by Montoro et al., 2020; ≤1.25 kg by Beaulieu et al., 2012; He et al., 2016, Douglas et al., 2014), although the results regarding subsequent growth performance remain controversial. Our findings align with studies by Huting et al. (2018), Zeng et al. (2019), Surek et al. (2019), and Montoro et al. (2020), which reported that light BIW piglets are capable of catching up to their heavier counterparts, achieving comparable BW by the end of the production cycle. Conversely, our results differ from those of Quiniou et al. (2002), Douglas et al. (2013), and He et al. (2016), who concluded that lightweight pigs at birth exhibit inferior growth performance compared to their heavier counterparts. Weights recorded at weaning and at the end of the nursery period have been shown to significantly influence subsequent growth performance. Thus, pigs with lower BW at later stages of the production cycle are generally expected to reach market weight later than their faster-growing counterparts (Douglas et al., 2014; Collins et al., 2017; López-Vergé et al., 2018). These findings suggest that farmers would benefit from systematically weighing animals at different stages of production to maintain a robust and updated database, which would be useful for efficiently identifying lighter animals. Interestingly, the importance of weight records along the specificity space increased notably for high specificity values. Therefore, weighing animals is crucial when implementing time-consuming individual treatments or expensive supplements, as these strategies should focus on truly underweight pigs, thereby avoiding false positives (i.e., high specificity).

It should be noted that incorporating additional prediction factors beyond BW into the analytical models could slightly improve AUC estimates, as these factors may influence the risk of a pig being slow-growing. One of the main factors influencing piglet growth performance during the lactation period is competition with littermates (Blavi et al., 2021; Riddersholm et al., 2021). In this context, litter size and the difference between birth BW and the average litter weight after cross-fostering appeared as the second and third most important factors, respectively, in classifying pigs at weaning. The within-litter weight variance also emerged as a main prediction factor during early growth stages, highlighting the importance of implementing effective management strategies such as cross-fostering (Patience et al., 2004). Additionally, practices like segregating piglets by BW or gender can help minimize subsequent weight variability, promoting more uniform growth within the batch. When classifying pigs at the end of the nursery period, birthweight-related factors were replaced not only by the WW but also by the season of birth. Interestingly, the season of birth demonstrated greater predictive significance for BW at the end of the nursery period than for WW. Douglas et al. (2013) highlighted the role of seasonality, suggesting that the month of birth can influence subsequent growth performance. Similarly, Paredes et al. (2012) identified the season of birth as one of the most important factors influencing the BW of pigs at the end of the nursery phase. The prediction of slaughter BW was strongly influenced by the BW at the end of the nursery period, resulting in the model achieving the highest AUC estimates across different production phases, as noted by Casellas et al. (2024). These results are consistent with the findings of López-Vergé et al. (2018), who observed that the probability of light pigs being sent to the slaughterhouse later increases as they approach the end of the production cycle.

In this study, generalized boosted regression and random forest consistently ranked highest in classification performance across all analyses. However, it is important to be careful when extrapolating these results to other datasets, as the performance of machine learning algorithms can vary significantly depending on the specific application and data types involved. The inclusion of additional prediction factors in the models could potentially enhance the classification ability of the algorithms. In our case, some limitations of the models were the inclusion of data from a single farm and only features up to weaning. However, as noted by Casellas et al. (2024), factors beyond the BW of pigs at each production stage may influence the risk of being slow-growing, but their impact on the analysis tends to be marginal, as the weight factors capture most of the critical information within the specificity space.

From a practical standpoint, we recommend evaluating a range of candidate algorithms (i.e., linear model, random forest, generalized boosted regression) for each application, rather than relying solely on a single approach. Once the best model is adjusted to a specific pig population and breed, the algorithm could be regularly updated without needing to monitor the entire population. Instead, updates could be based on sentinel animals within each batch. This approach could be highly useful for both decision-making within the herd and assessing the impact of specific management practices and nutritional interventions (i.e., cross-fostering, ensuring adequate colostrum intake, and providing creep-feed, among others) aimed at target variables, such as reducing the risk of low BW at various stages of the production system. Notably, these interventions are particularly effective during the early growth phases, especially during the lactation period, which has been identified as a critical window for influencing pigs’ BW category and enhancing the growth performance of lighter piglets (Blavi et al., 2021).

## CONCLUSION

In conclusion, our study demonstrates that machine learning algorithms are reliable and effective tools for classifying commercial pigs at risk of growth retardation across different production stages. Both random forest and generalized boosted regression models exhibited the highest classification performance. Their applicability in commercial herds may form the basis for decision-making on management strategies in the swine industry.

## Abbreviations

AUC: area under the curve
BIW: birth weight
BW: body weight
DB: difference between the piglet’s birth weight and the average litter weight at birth
DC: difference between the piglet’s birth weight and the average litter weight after cross-fostering
ROC: receiver operating characteristic
VB: within-litter variance of body weight at birth
VC: within-litter variance of body weight after cross-fostering
WW: weaning weight
